# Celastrol Loaded Nanoparticles With ROS-Response and ROS-Inducer for the Treatment of Ovarian Cancer

**DOI:** 10.3389/fchem.2020.574614

**Published:** 2020-10-30

**Authors:** Weina Niu, Jianguo Wang, Qinyao Wang, Jianjun Shen

**Affiliations:** ^1^Department of Oncology, The First Affiliated Hospital of USTC, Division of Life Sciences and Medicine, University of Science and Technology of China, Hefei, China; ^2^Department of Radiation Oncology, The First Affiliated Hospital of USTC, Division of Life Sciences and Medicine, University of Science and Technology of China, Hefei, China

**Keywords:** ovarian cancer, celastrol, PLGA-TK-PEG-FA, oxidative stress amplification, ROS-response, ROS-inducer

## Abstract

Ovarian cancer is a gynecological cancer from which it is difficult to be completely cured. It is common to use regimens as an effective treatment for ovarian cancer, but these inevitably bring serious side effects. New treatment strategies and special drugs are needed to improve the prognosis of patients. Celastrol is a natural product, isolated from traditional medicine, that has been proven to be curative for inflammation and cancers. However, the non-targeting and low solubility of celastrol limit its clinical application. We prepared celastrol-loaded nanoparticles for the efficient treatment of ovarian cancer via oxidative stress amplification. In this work, a tumor-targeted, ROS-sensitive nanoparticle was designed, synthesized, and assembled into a drug delivery system that used celastrol. Folic acid (FA) groups on the surface of nanoparticles guide them to actively target the surface of the tumor cell membrane. Thioketal (TK) bonds in nanoparticles can be oxidized and broken into -SH within the ROS level of tumor tissues, which causes the breaking of the PEG hydrophilic shell layer of nanoparticles and promotes the release of celastrol. The released celastrol further stimulated the production of ROS and amplified the intracellular ROS level to promote the apoptosis of tumor cells, thus achieving a therapeutic effect on the celastrol treated ovarian cancer.

## Introduction

Ovarian cancer is a fatal gynecological cancer, and aggressive surgical treatment combined with chemotherapy still cannot guarantee a good prognosis.

At present, the adjuvant treatment of ovarian cancer generally depends on the patient's condition, such as CP (cyclophosphamide + cisplatin), TP (Taxol + carboplatin), and CAP (cyclophosphamide + Adriamycin + Cisplatin). Although the mentioned therapeutic schedules are effective in the treatment of ovarian cancer, they can cause significant side effects in the patient, and new agents that are capable of improving the prognosis of ovarian cancer are urgently needed (Gallardo-Rincon et al., 2016; Chen et al., 2018).

Traditional medicine plays an important role in the treatment of diseases, and the natural products from traditional medicine feature a diversity of structures and targets that are an important source in the development of new drugs. Celastrol derives from *Tripterygium wilfordii* and has a wide range of biological and pharmacological activities.

A study by Trott et al. initially found that celastrol significantly inhibits the activity of HSP90 (Heat Shock Proteins) by promoting nuclear transport of HSF1 (Heat Shock Transcriptional Factor 1) (Trott et al., 2008). Subsequently, a large number of scientists have carried out in-depth research on celastrol. In recent years, celastrol has been found to have significant antitumor properties independent of HSP90. Celastrol has been found to induce programmed cell death by activating glycogen synthase kinase 3β (Feng et al., 2013). Treatment of celastrol inhibits cancer growth by activating the TNF- α-induced NF-κB signaling pathway (Kang et al., 2013). Moreover, it induces the apoptosis of carcinoma cells by the WNT/β-catenin pathway (Lu et al., 2012), and inhibits the proliferation and invasion of colorectal cancer by repressing the epithelial-mesenchymal transformation (EMT) (Divya et al., 2018; Wang et al., 2019). Multiple studies have shown that celastrol induces the increase of ROS levels and induces intracellular accumulation of ROS to promote cell apoptosis in a variety of tumor cells such as melanoma (Lee et al., 2012), liver cancer (Liu et al., 2016), glioma (Liu et al., 2019), and osteosarcoma (Li et al., 2015), etc.

Unfortunately, celastrol had many properties that prevented it from being used as a therapeutic agent. Celastrol had a very strong hydrophobicity, poor corresponding bioavailability, and short serum half-life (T1/2β). The clinical application of celastrol is still limited by its narrow therapeutic window and poor solubility (Wang et al., 2016; Chen et al., 2018). Celastrol also had many targets unrelated to tumor surface receptors and affected by a variety of cell types, which meant poor targeting performance to the tumor *in vivo*.

Nanomaterials have been designed to solve each of these problems. For example, the encapsulation and transport of celastrol in nanocarriers enhanced its water solubility, changed its biological distribution and serum half-life. Nanocarriers reduced off-target effects by targeting specific cells or tissues to increase the selectivity of encapsulated celastrol. These benefits were to reduce the effective dose of significant therapy, minimize toxicity, and improve safety.

In this work, a tumor-targeted, ROS-sensitive nanoparticle was designed, synthesized, and assembled into the drug delivery system of celastrol. Folic acid (FA) groups on the surface of nanoparticles were used to guide the nanoparticles to actively target the surface of the tumor cell membrane. Thioketal (TK) bonds in nanoparticles were oxidized and broken into -SH within the ROS level (20–200 μM) of tumor tissues, which caused the breaking of the PEG hydrophilic shell layer of nanoparticles and promoted the release of celastrol. The released celastrol further stimulated the production of ROS and amplified the intracellular ROS level to promote the apoptosis of tumor cells, thus achieving a therapeutic effect on the celastrol treated ovarian cancer.

## Experimental Materials

Boc-PEG_2k_-NH_2_, PLGA_5k_, polyvinyl alcohol (PVA), PLGA_5k_-mPEG_2k_, were purchased from HWRK CHEM Co., Ltd. (Beijing, China). Acetone, 3-mercaptopropionic acid, trifluoroacetic acid (TFA), N, N-Dimethylformamide (DMF), Dichloromethane (DCM), anhydrous ether obtained from Energy chemical Inc. Celastrol, NaCl, NaHCO_3_, 4-dimethylaminopyridine (DMAP), N, N-Dicyclohexylcarbodiimide (DCC), folic acid (FA), fluorescein isothiocyanate isomer I (FITC), rhodamine-B (RhB), penicillin, streptomycin and 4′, 6-diamidino-2-phenylindole (DAPI) were purchased from Aladdin Inc. Dulbecco's modified Eagle's medium (DMEM), 100 × mycillin, and fetal bovine serum (FBS) were purchased from Gibco Inc.

Ovarian cell lines SKOV3 were purchased from BeNa culture Collection, all cell experiments complied with the ethical review of animal experiments in The First Affiliated Hospital of USTC.

## Methods

### Synthesis of TK

Under the protection of nitrogen, a solution of acetone (0.50 g, 2.77 mM), 3-mercaptopropionic acid (0.36 mL, 4.16 mM), and a catalytic amount of TFA were stirred at room temperature for 12 h. A saturated NaHCO_3_ extraction solution was used to quench the reaction. The precipitate was collected by filtration and was washed with cold water. The obtained white powder was recrystallized from hexane and dried in a vacuum oven overnight.

### Synthesis of Boc-PEG_2k_-TK

Under the protection of nitrogen, Boc-PEG_2k_ -NH_2_ (2.4 g), DMAP (0.012 g, 0.1 mM) and TK (0.374 g, 1 mM) dissolved in cold DMF (25 mL) under nitrogen protection. DCC (0.412 g, 2 mM) was dissolved in cold DMF (5 mL) and added to the mixed solution drop by drop, stirring at room temperature for 48 h. The resulting white precipitate was filtered and the concentrated filtrate reprecipitated in cold anhydrous ether. The precipitation was redissolved in 1 mL DCM and precipitated in cold anhydrous ether to obtain the precipitation of the product.

### Synthesis of PLGA_5k_-TK-PEG_2k_-NH_2_

Boc-mPEG_2k_ -TK (2.4 g), PLGA_5k_ (7.5 g), and DMAP (0.012 g, 0.1 mM) were dissolved in 25 mL DMF in an ice bath under nitrogen atmosphere. DCC (0.412 g, 2 mM) was dissolved in DMF (5 ml), added drop by drop to the mixing system, and stirred at room temperature for 48 h. The resulting white precipitate was filtered and the concentrated filtrate was reprecipitated in the cold anhydrous ether. The precipitate was further redissolved in 5 mL DCM, with a 5 mL TFA drop in. It was then stirred at room temperature for 2 h, and the mixture was extracted by saturated Na_2_CO_3_ and NaCl in turn. The resulting solution was concentrated and reprecipitated in the cold anhydrous ether.

The obtained precipitate was further dissolved in the 5 mL DMF and extensively dialyzed (MWCO 7 kDa, Spectrum Laboratories, Laguna Hills, CA) against deionized water.

**Figure 1:**
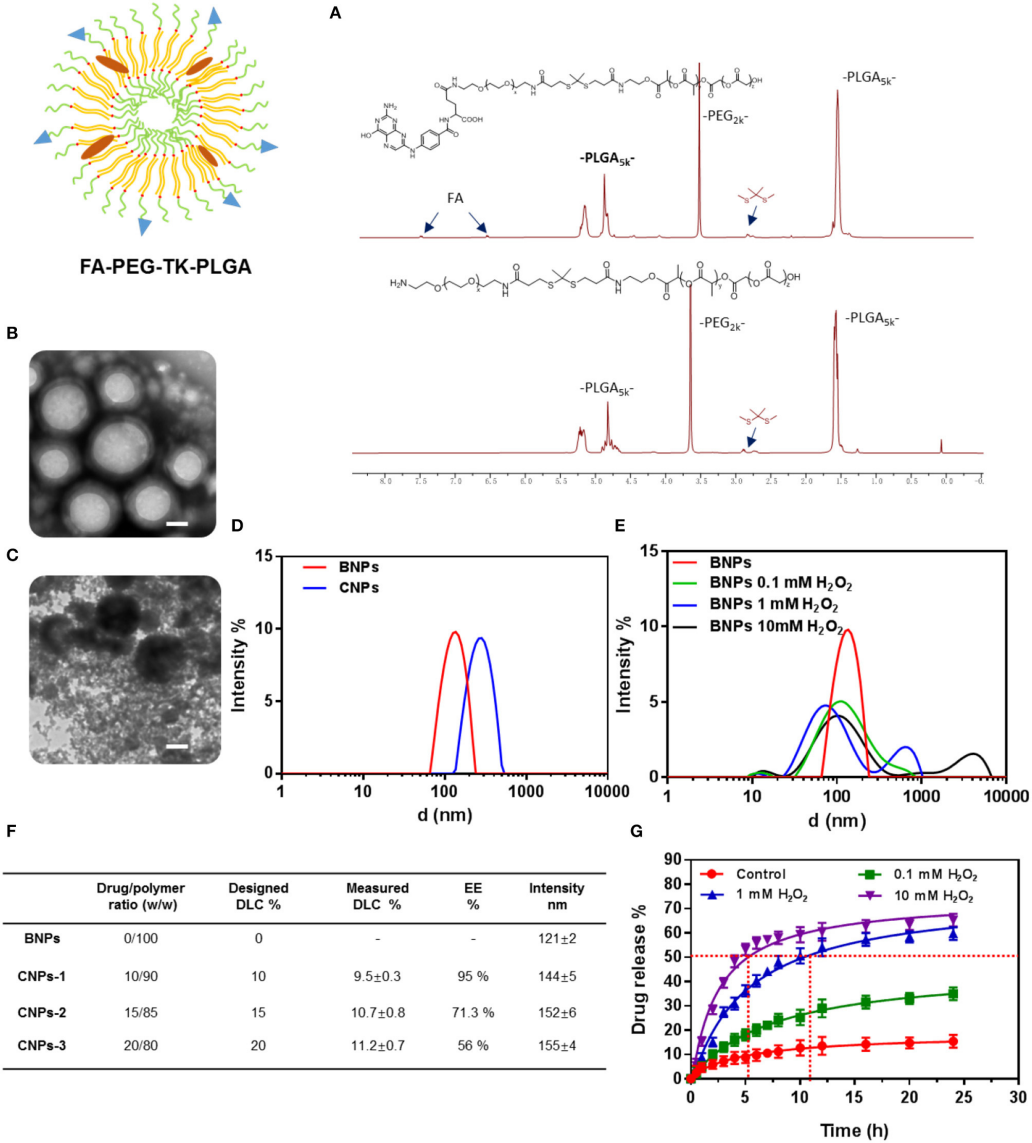
NMR 1H of PLGA-TK-PEG-NH2 and PLGA-TK-PEG-FA **(A)**; TEM characterization of BNPs **(B)** and BNPs stimulated by ROS (1 mM H_2_O_2_, 20 μM CuCl_2_) **(C)** Scale bar: 50 nm; DLS of BNPs and Cel-loaded nanoparticles **(D)** and nanoparticles stimulated by ROS (0.1, 1, 10 mM H_2_O_2_, 20 μM CuCl_2_) **(E)**; DLC and EE of two amphiphiles **(F)**; drug release rate of Cel@FA-NPs **(G)**.

The product PLGA_5k_ -TK-PEG_2k_ -NH_2_ was vacuum-dried at 25°C for 6 h (yield = 79%) and characterized by 1 H NMR (Figure 1A).

### Synthesis of PLGA_5k_-TK-PEG_2k_-FA

PLGA_5k_-TK-PEG_2k_-NH2 was further modified with FA. The specific method was to dissolve FA (0.088 g, 2 mM), DMAP (0.012 g, 0.1 mM), and DCC (0.412 g, 2 mM) in 30 mL cold DMF of under the protection of nitrogen. After activation for 30 min, a 5 mL DMF solution containing 1 g PLGA_5k_-TK-PEG_2k_-NH_2_ was added into the mixture drop by drop, and the reaction continued for 24 h at room temperature. After the white precipitate was filtered, the concentrated filtrate reprecipitated in the cold n-hexane. The product PLGA_5k_-TK-PEG_2k_-FA was vacuum-dried at 25°C for 6 h (yield = 79%) and characterized by 1 H NMR (Figure 1A).

### Preparation of BNPs and CNPs

A total of 500 μL PLGA_5k_-TK-PEG_2k_-FA solution (100 mg/mL, chloroform) was added to 6 mL of 1% PVA. In the ice bath, the ultrasonic probe was used for 5 min (80 W) to form an oil-water emulsion. The mixture was added to 30 mL 0.3% PVA and stirred overnight to volatilize chloroform and solidify the surface of the PLGA ball. The ultrafiltration concentration was cleaned with a 100 KD ultrafiltration tube. The volume was fixed to 5 mL with pure water, and the mother solution of BNPS was collected and stored at 4°C. Other different concentrations of BNPs were obtained by the dilution of the mother solution.

The celastrol was dissolved in chloroform and prepared into a solution of 20 mg/mL.

A total of 500 μL PLGA_5k_-TK-PEG_2k_-FA solutions (50 mg/mL, chloroform) and 50 μL of celastrol (20 mg/mL, chloroform) was added to 6 mL of 1% PVA ultrasonicated for 5 min by the ultrasonic probe (80 W) to form an oil-water emulsion. The mixture was added to 30 mL 0.3% PVA and stirred overnight to volatilize chloroform and solidify the surface. The ultrafiltration concentration was cleaned with 100 KD ultrafiltration tube. The volume was fixed to 5 mL with pure water, and the mother solution was collected and stored at 4°C. Other different concentrations of CNPs were obtained by the dilution of the mother solution.

### TEM Characterizations

Morphology of BNPs and CNPs were examined by transmission electron microscopy. The carbon disc ultrathin grid was ionized under vacuum (0.3 Torr, 400 V, 20 s). Imaging was performed at a tension of 100 keV and a magnification at 30,000 × (Hitachi, HT7800).

### DLS

The particle size of the BNPs and CNPs were determined by dynamic light scattering (DLS). Samples were measured at a polymer concentration of 5 mg/mL and were equilibrated at 25°C for 60 s before the first measurement. Particle size and distribution were obtained as triplicate and expressed as size distribution by number.

Fresh PBS solutions (pH 6.5), containing 10 mM H_2_O_2_ and 3.2 μM CuCl_2_, were prepared to simulate the ROS conditions. The size changes of the nanoparticles in response to ROS conditions were measured by DLS (3000HS, Malvern Instruments Ltd.)

In brief, 1 mL of the solution of the nanoparticles (1 mg/mL) was diluted with 1 mL of PBS (pH 7.4) containing different concentrations of ROS. The solution was then incubated at 37°C in a thermostatic water bath oscillator and the size was measured at a predetermined time interval by DLS.

### DLC and EE

The encapsulation efficiency (EE) and drug load capacity (DLC) of celastrol were evaluated by high performance liquid chromatography (HPLC) using Thermo Scientific C18 reversed phase column with a mobile phase of DMF at 0.5 mL/min.

The celastrol absorption curve at 280 nm was quantified and the standard curve of celastrol was established, demonstrating good linearity from 12 μg/mL to 2 mg/mL.

The lyophilized amphiphilic polymers (8.5 mg) and celastrol (1.5 mg) were dissolved in 2 mL of DMF and assembled into CNPs according to the method above. A total of 1 mg lyophilized CNPs were redissolved in 200 μL DMF, and the content of celastrol was quantitatively determined by HPLC.

The encapsulation efficiency (EE) and drug loading capacity (DLC) of celastrol were calculated by the following formula:

$$\text{DLC( ​​}\%\text{​​ )=}\left\{\frac{\text{drug weight in drug-loaded micelles}}{\text{weight of drug-loaded micelles}}\right\}\times \text{100 ​​}\%\text{​​ }$$

$$\text{EE( ​​}\%\text{​​ )=}\left\{\frac{\text{drug weight in drug-loaded micelles}}{\text{weight of drug in feeding}}\right\}\times \text{100 ​​}\%\text{​​ }$$

### Release Profiles of Drug-Loaded NPs

One milliliter CNPs were put into a dialysis bag (3.5 kda, CA), and the drug release characteristics of CNPs were studied in PBS buffer containing H_2_O_2_ (0.1 mM, 1 mM, 10 mM) and CuCl_2_ (20 μM). The content of celastrol outside the dialysis bag was concentrated and measured at a predetermined time interval by HPLC.

### Cell Culture

Ovarian cancer cell (SKOV3) was purchased from the Cell Bank of the Chinese Academy of Science (Shanghai, China). 1640 medium supplemented with 10% fetal bovine serum (FBS), 1% penicillin/streptomycin, 1.5 g/L sodium carbonate, and 0.11 g/L sodium pyruvate was used to culture SKOV3 cells in a humid atmosphere with 37 °C and 5% CO_2_.

### Cell Viability

SKOV3 cells were inoculated into 96-well plates (20,000 cells per well). The cells were then treated with free celastrol, BNPs, and CNPs. They were then incubated with free celastrol, BNPs, and CNPs for 24 h, and the cells were washed and cell viability was measured by CCK8 assay. The absorbance at 450 nm was determined by a microplate reader (Molecular Devices, Sunnyvale, CA, USA).

### Wound Healing Assay

SKOV3 cells were seeded into 6-well plates (50,000 cells per well) for 24 h. Cells were then scratched by a 200 μL pipette tip and cultured with free celastrol, BNPs, and CNPs for another 24 h. The scratch changes were photographed by an optical microscope.

### Fluorescence Imaging

SKOV3 cells were seeded into 6-well plates (20,000 cells per well), incubated with PBS, free celastrol, BNPs, FL-BNPs, FL-CNPs for 24 h. NAC was added to balance intracellular ROS and to verify the effect of intracellular ROS on cell phagocytosis.

After being washed with serum-free MEM, the cells were observed by laser confocal microscopy (Leica, Wetzlar, Germany).

### Reactive Oxygen Analysis

SKOV3 cells were seeded into 6-well plates (25,000 cells per well), incubated with PBS, free celastrol, BNPs, CNPs for 24 h. The cells were then incubated with 10 μM DCF-DA at 37°C for 30 min. Washed with serum-free MEM, the intracellular ROS levels were observed by fluorescence microscopy (Leica, Wetzlar, Germany) and measured by Microplate Reader (BD Biosciences, San Jose, California, USA).

### Cell Apoptosis

SKOV3 cells were seeded into 6-well plates (50,000 cells per well) and incubated with different interventions for 24 h. The apoptosis effect was measured via an Annexin V-FITC/PI apoptosis detection kit (Beyotime Biotech, China) by flow cytometer.

## Results and Discussions

### Characterization of Amphiphilic Polymers and Nanoparticles

Characterization of the polymer was obtained by ^1^H NMR. The nuclear magnetic characterization of polymers after solvent peaks as shown in Figure 1A. The characteristic TK bond peaks of PLGA_5k_-TK-PEG_2k_ were observed at 2.8 ppm. For PLGA_5k_-TK-PEG_2k_-FA, the characteristic peaks of FA at 6.7 and 7.5 ppm could be observed.

The CNPs with stable particles, uniform size, and average particle size of 120 nm were prepared by reverse evaporation. The morphology of the nanoparticles was characterized by TEM. The uniform spherical hydrophobic core of nanoparticles had a ring of faint hydrophilic shells (Figure 1B). A large number of PEG segments were cut off by stimulating the TK bond fracture with ROS after the hydrophilic shell layer of nanoparticles was detached. After the ROS-stimulated nanoparticles were stained with phosphotungstic acid, it was observed that a large number of PEG fragments were dispersed in the solution, while the PLGA chain segments were aggregated into black nanoparticles of different sizes due to hydrophobic action (Figure 1C).

The particle size of the prepared nanoparticles was further measured by the Dynamic light scattering, as shown in Figure 1D (DLS, Santa Barbara, Nicomp 380 ZLS).

The solution with H_2_O_2_ (0.1, 1, 10 mM) and catalytic CuCl_2_ was prepared to simulate the ROS solution. BNPs were mixed with ROS solution for 2 h and measured by the Dynamic light scattering. The results were shown in Figure 1E. When the concentration of H_2_O_2_ increased from 0.1 to 10 mM, the proportion of the particle size at 100 nm gradually decreased. When the concentration of H_2_O_2_ reached 1 mM, a significant secondary peak appeared at 700 nm. When the concentration of H_2_O_2_ was further increased to 10 mM, the hydrophobic nanoparticle aggregation effect was more obvious, and the sub-peak position appeared at 3,000 nm. A large number of tiny particles appeared after ROS stimulation. This phenomenon was not presented in the DLS results, compared with TEM images (Figure 1C). It was ascribed to the fact that the PEG segments were hydrophilic and did not aggregate into particles in the solution.

As shown in Figure 1F, the average particle size of the BNPs was 121 nm. After celastrol was encapsulated into nanoparticles, the average particle size of CNPs increased to 155 nm. The entrapment efficiency (EE) and drug loading capacity (DLC) of nanoparticles were measured by HPLC, as shown in the table (Figure 1F). As the designed DLC gradually increased from 10 to 20%, the measured DLC increased from 9.5 to 11.2%, while the EE decreased from 95 to 56%.

The drug release characteristics of CNPs in a tumor cell microenvironment were simulated by simulating the environment with different ROS concentrations, as shown in Figure 1G.

9.4% celastrol was released from the CNPs in the PBS solution within 24 h. A low concentration of ROS (0.1 mM) stimulated the release of celastrol, and the release rate of celastrol increased from 9.4 to 31.7% within 24 h. Medium concentration of ROS (1 mM) further increased the release rate from 31.7 to 59.6%, while high concentration of ROS (10 mM) increased the release rate very little (59.6–62.3%).

At the same time, it can be seen from Figure 1G that the higher the ROS concentration was, the faster celastrol would be released. In high-concentration ROS solutions, Celastrol's release rate reached more than 50% within just 5 h. To achieve the same release rate, a medium concentration of ROS solution needed 11.5 h.

**Figure 2:**
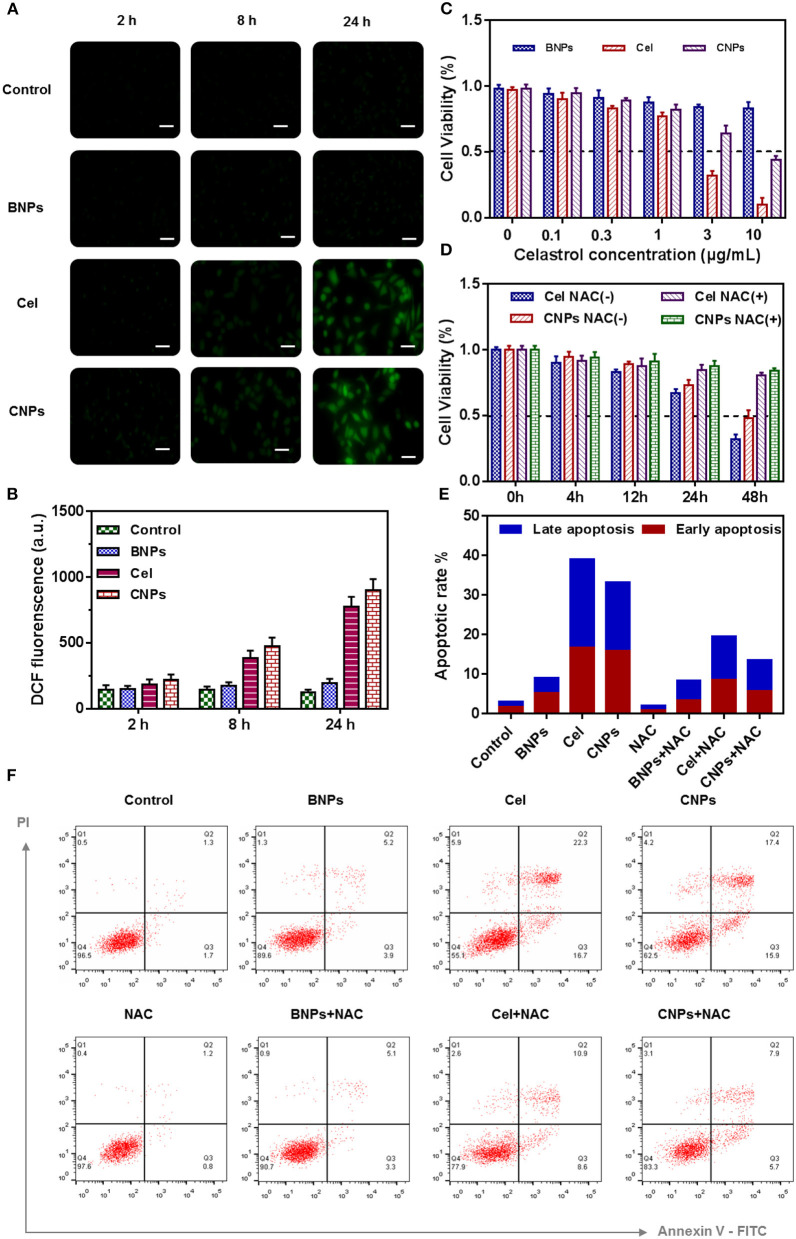
Evaluation of the ROS regenerating ability of PBS, BNPs, celastrol, and CNPs *in vitro*. Fluorescence images of SKOV3 cells treated with different micelles for different time **(A)**. Scale bar: 20 μm. Quantitative analysis of the ROS generation in SKOV3 cells by a microplate reader **(B)**. Cell viability **(C,D)**, Apoptosis analysis **(F)**, and Quantification of apoptosis result **(E)** of SKOV3 cells induced by PBS, BNPs, celastrol, and CNPs. All error bars were presented as mean ± SD.

SKOV3 cells were incubated with PBS, free Celastrol, BNPs, and CNPs for 24 h to obtain the results of cell viability, as shown in Figure 2.

In Figures 2C,D the cell viability of SKOV3 was determined by CCK8 24 h after different drug treatments. The dissociated celastrol gradient concentration (0, 0.1, 0.3, 1, 3, 10 mM) was set, and the corresponding celastrol loaded NPs were configured according to the equivalent concentration, while mass concentrations such as BNPs and celastrol loaded NPs were configured.

BNPs were less toxic to SKOV3, as the concentration reached 10 μg/mL, and the cell survival rate was up to 87%. Free celastrol was highly cytotoxic to SKOV3. When the concentration of celastrol reached 1 μg/mL, the cell survival rate dropped to <80%. When the celastrol concentration reached 10 μg/mL, the cell survival rate was only 18.2%. According to the cell survival curve, the IC50 value of celastrol was estimated to be 1.4 μg/mL. The cytotoxicity of CNPs was also cytotoxic to SKOV3, but the toxicity of CNPs was slightly lower than that of free celastrol. When the concentration of CNPs reached 10 mM, the cell survival rate of SKOV3 was 48.3%. We speculated that celastrol wrapped in nanoparticles was not fully released during the 24-h cell culture process, and this feature was also combined with the drug release characteristics of CNPs.

NAC is a ROS scavenging agent and was used to balance intracellular ROS stress. NAC (10 mM) was used in combination with free celastrol and CNPs to evaluate the toxic effect of high ROS on SKOV3 cells, as shown in Figure 2D. Apparently, without NAC intervention, free celastrol and CNPs significantly reduced SKOV3 cell activity within 48 h, while NAC intervention increased the cell activity to more than 80%.

SKOV3 cells were incubated with PBS, BNPs, celastrol, and CNPs for 24 h, intracellular ROS were labeled with the ROS dye DCF-DA (2 mM). By labeling with DCF-DA, the intracellular ROS level was further characterized and measured after treatment with different drugs. As shown in Figures 2A,B free celastrol and CNPs treatment significantly improved the level of oxidative stress in SKOV3 cells, and the longer the treatment period was, the higher the intracellular ROS level presented, which suggested that celastrol induced cells to produce high levels of ROS.

Further apoptosis experiments were conducted to verify the apoptosis-inducing effect of drugs on cells and the significant influence of NAC on drug treatment, as shown in Figure 2F.

**Figure 3:**
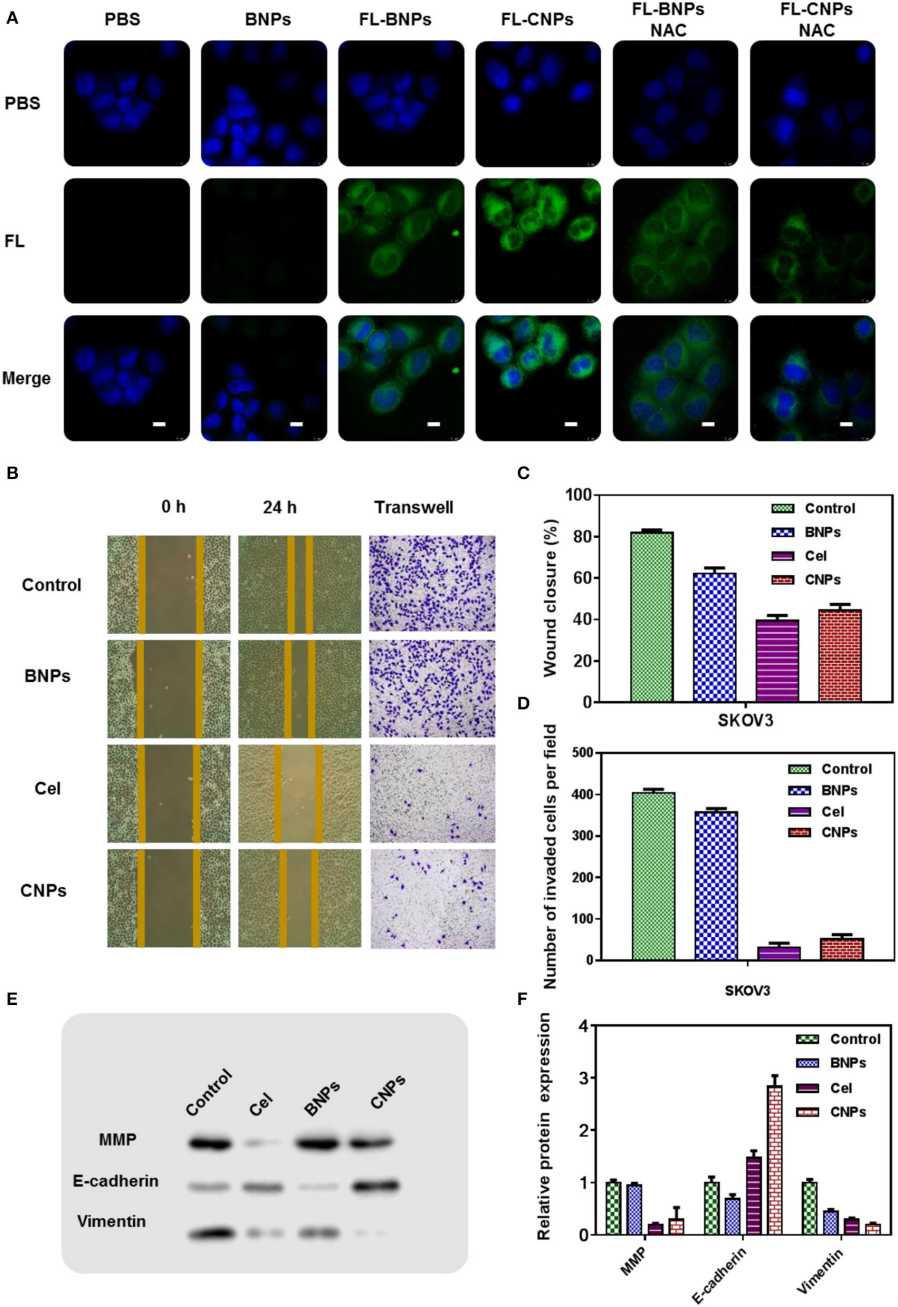
Confocal laser scanning microscopy (CLSM) images of SKOV3 treated with PBS, BNPs, FL-BNPs, and FL-CNPs, compared with NAC intervention, Scale bar: 10 μm **(A)**; Wound healing **(B,C)**, tunnel **(B,D)**, and the protein expression **(E,F)** of SKOV3 treated with PBS, BNPs, celastrol, and CNPs.

After SKOV3 cells were incubated with PBS, BNPs, FL-BNPs, and FL-CNPs for 24 h, the nuclei were labeled with DAPI dye. The cell fluorescence was photographed by laser confocal imaging, as shown in Figure 3A.

Significant green fluorescence was observed after FL-BNPs and FL-CNPs treated cells. Notably, FL-CNPs exhibited stronger green fluorescence than FL-BNPs.

When NAC was combined with different drug treatments, significant fluorescence attenuation appeared in FL-BNPS&NAC treatment and FL-CNPS&NAC treatment, which suggested that intracellular ROS levels significantly affected the phagocytosis of nanomaterials at the cellular level.

We hypothesize that, although the TK bond in nanoparticles not only consumed intracellular ROS, it also consumed intracellular GSH, reducing GSH levels. After the cells swallowed the nanoparticles, the nanoparticles would stimulate the production of intracellular ROS. In general, the treatment of BNPs increased the intracellular ROS level.

The experimental results of scratch healing and TUNEL (Figures 3B–D) showed that without any intervention, the cell scratch healing was 80% 24 h later, and the wound healing of SKOV3 in the BNPs group was 65%, slightly less than that in the control group. The cell wound healing in the celastrol treatment group was 41%, while the cell scratch healing in the CNPs group was 46%. The cell healing ability in the celastrol treatment group and the CNPs group was far less than that in the control group, indicating that they could effectively inhibit the cell migration and invasion of SKOV3. The same conclusion was obtained from the TUNEL experiment results.

The expression of proteins associated with oxidative stress was further detected by WB. Specifically, SKOV3 cells were incubated with PBS, BNPs, celastrol, and CNPs for 24 h. Proteins were extracted after cell fragmentation for western blot (WB) detection to detect the contents of matrix metalloproteinases (MMP), E-calcine, and Vimentin in cells of different treatment groups (Figures 3E,F was the numerical statistical result of WB). The matrix metalloproteinases (MMP), E-cadherin, and vimentin EMT-related proteins determined the aggressiveness of tumor cells.

WB results showed that after celastrol and CNPs treatment, E-cadherin was significantly up-regulated, while matrix metalloproteinases and vimentin were down-regulated. This suggested that celastrol and CNPs treatments effectively inhibited epithelial mesenchymal transformation.

## Conclusion

In this work, a tumor-targeted, ROS-sensitive nanoparticle was designed, synthesized, and assembled into the drug delivery system of celastrol. Folic acid (FA) groups on the surface of nanoparticles guide the nanoparticles to actively target the surface of the tumor cell membrane. Thioketal (TK) bonds in nanoparticles were oxidized and broken into -SH within the ROS level (20–200 μM) of tumor tissues, which caused the breaking of the PEG hydrophilic shell layer of nanoparticles and promoted the release of celastrol. The released celastrol further stimulated the production of ROS and amplified the intracellular ROS level to promote the apoptosis of tumor cells, thus achieving a therapeutic effect on the celastrol treated ovarian cancer.

Our experimental results indicate an effective strategy of inducing tumor cell apoptosis by delivering ROS sensitive nanomaterials to tumor cells and accelerating the release of natural product drugs that stimulated cells to further produce ROS, using the high ROS microenvironment in tumor cells. The results provide a theoretical basis for further animal experiments.

## Data Availability Statement

The raw data supporting the conclusions of this article will be made available by the authors, without undue reservation.

## Author Contributions

This project was conceptually designed by JS. The majority of the experiments were performed by WN and JW, assisted by QW. Data analysis and interpretation were carried out by WN and JS. This manuscript was prepared by JS. All authors discussed the results and commented on the manuscript. All authors contributed to the article and approved the submitted version.

## Conflict of Interest

The authors declare that the research was conducted in the absence of any commercial or financial relationships that could be construed as a potential conflict of interest.
